# Development of α-Synuclein Real-Time Quaking-Induced Conversion as a Diagnostic Method for α-Synucleinopathies

**DOI:** 10.3389/fnagi.2021.703984

**Published:** 2021-09-28

**Authors:** Takehiro Nakagaki, Noriyuki Nishida, Katsuya Satoh

**Affiliations:** ^1^Department of Molecular Microbiology and Immunology, Nagasaki University Graduate School of Biomedical Sciences, Nagasaki, Japan; ^2^Department of Health Sciences, Unit of Medical and Dental Sciences, Nagasaki University Graduate School of Biomedical Sciences, Nagasaki, Japan

**Keywords:** α-synuclein (α-syn), Parkinson’s disease, dementia with Lewy bodies (DLB), antemortem diagnosis, RT-QuIC, multiple system atrophy (MSA)

## Abstract

Parkinson’s disease, dementia with Lewy bodies, and multiple system atrophy are characterized by aggregation of abnormal α-synuclein (α-syn) and collectively referred to as α-synucleinopathy. Because these diseases have different prognoses and treatments, it is desirable to diagnose them early and accurately. However, it is difficult to accurately diagnose these diseases by clinical symptoms because symptoms such as muscle rigidity, postural dysreflexia, and dementia sometimes overlap among these diseases. The process of conformational conversion and aggregation of α-syn has been thought similar to that of abnormal prion proteins that cause prion diseases. In recent years, *in vitro* conversion methods, such as real-time quaking-induced conversion (RT-QuIC), have been developed. This method has succeeded in amplifying and detecting trace amounts of abnormal prion proteins in tissues and central spinal fluid of patients by inducing conversion of recombinant prion proteins *via* shaking. Additionally, it has been used for antemortem diagnosis of prion diseases. Recently, aggregated α-syn has also been amplified and detected in patients by applying this method and many clinical studies have examined diagnosis using tissues or cerebral spinal fluid from patients. In this review, we discuss the utility and problems of α-syn RT-QuIC for antemortem diagnosis of α-synucleinopathies.

## Introduction

α-Synucleinopathies (α-synucleinopathies) are disorders characterized by aggregation and deposition of α-synuclein (α-syn), which include Parkinson’s disease (PD), dementia with Lewy bodies (DLB), and multiple system atrophy (MSA). PD was first described by British physician James Parkinson in 1817 (Tysnes and Storstein, 2017). PD patients present with motor and autonomic impairments, especially parkinsonisms such as rigidity, rest tremor, bradykinesia, and postural dysreflexia. DLB was first reported by Kosaka (1978) in the late 1970s and is characterized by progressive dementia, visual hallucinations, and parkinsonism (Donaghy and McKeith, 2014); it is the second most common cause of dementia in Japan after Alzheimer’s disease (AD). Histopathological analysis has revealed neuronal loss and Lewy bodies (LBs) in the cerebrum and brainstem of patients (Spillantini et al., 1997; Baba et al., 1998). Because LBs have been commonly seen in both PD and DLB, these diseases are pathologically named Lewy body diseases (LBDs). However, MSA is a collective term for olivopontocerebellar atrophy, striatonigral degeneration, and Shy–Drager syndrome (Kaji et al., 2020) because of the similarities in symptoms and pathology. Papp et al. (1989) and Nakazato et al. (1990) identified lethargic inclusions in the vacuoles of oligodendroglia in MSA and described the appearance of glial cytoplasmic inclusions (GCIs) in all subtypes of these three diseases. MSA patients present with autonomic symptoms such as dysuria, orthostatic hypotension (OH), and erectile dysfunction, and they are classified as MSA with predominant cerebellar ataxia (MSA-C) and MSA with predominant parkinsonian features (MSA-P) (Gilman et al., 2008; Mitsui et al., 2015).

Because α-syn is a major component of LBs in PD/DLB patients and GCI in MSA patients (Spillantini et al., 1997; Wakabayashi et al., 1998), these diseases are now unified under the concept of α-synucleinopathy.

Accurate diagnosis is very important at an early stage of these diseases because deposition of pathological α-syn occurs before the symptoms of α-synucleinopathies. For example, it has been reported that 50–70% of dopaminergic neurons are already lost by the time PD is clinically diagnosed (Hughes et al., 1992). Additionally, parkinsonism in DLB has been reported to be less responsive to L-DOPA than that in PD (Lucetti et al., 2010). Thus, even in the same α-synucleinopathy, the treatment strategy and prognosis may differ depending on the disease. Moreover, diagnosis of α-synucleinopathy by relying on clinical symptoms is difficult because symptoms sometimes overlap between α-synucleinopathies and other neurodegenerative disorders including AD, progressive supranuclear palsy (PSP), corticobasal degeneration (CBD), and Creutzfeldt–Jakob disease (CJD) (Armstrong et al., 2013; Hoglinger et al., 2017; McKeith et al., 2017; Geschwind and Murray, 2018; Zerr and Hermann, 2018; Fabbrini et al., 2019). Hence, the most desirable method for preclinical diagnosis is direct detection of aggregated α-syn in patients.

*In vitro* conversion methods, such as protein misfolding cyclic amplification (PMCA) (Saborio et al., 2001; Castilla et al., 2006) and real-time quaking-induced conversion (RT-QuIC) (Wilham et al., 2010), have been developed in the field of prion research. These methods induce conversion of normal prion proteins in brain homogenates or recombinant prion proteins to abnormal prion proteins by sonication or shaking, which allows amplification and detection of trace amounts of abnormal prion proteins in tissues or body fluids. They have been reported by many clinical studies as a preclinical diagnostic method (Hermann et al., 2021).

Aggregated α-syn deposition has been reported in various peripheral tissues in patients with α-synucleinopathy, including the digestive system, skin, and salivary glands (Beach et al., 2010; Wang et al., 2013; Stokholm et al., 2016; Lee et al., 2017; Niemann et al., 2021), and in CJD patients (Takatsuki et al., 2016; Orru et al., 2017; Satoh et al., 2019). α-Syn RT-QuIC using cerebrospinal fluid (CSF), skin, or olfactory mucosa (OM) samples of patients has been developed similarly to prion RT-QuIC. Here, we review the most recent findings on α-syn RT-QuIC using CSF, skin, or OM samples.

## α-Synuclein and Real-Time Quaking-Induced Conversion

α-Synuclein is a presynaptic protein that consists of 140 amino acids, which can be divided into three domains: N-terminal, middle, and C-terminal domains. The N-terminal domain (aa 1–60) contains the highly conserved repeat KTKEGV and has an α-helix propensity. The middle domain (aa 61–95) contains a non-amyloid β component (NAC) region and shows a β-sheet propensity. The C-terminal domain (aa 96–140) is enriched with proline and negatively charged amino acids (glutamine and asparagine) and is an intrinsically disordered region that contributes to the maintenance of solubility (Breydo et al., 2012; Villar-Piqué et al., 2016; Wang et al., 2016). α-Syn has been reported to be a soluble and monomeric disordered protein in neurons, but has also been reported to form macromolecular assemblies and adopt a variety of structures. α-Syn aggregation is induced by deletion of its C-terminal domain. Hence, the interaction with the NAC region plays a crucial role in its structural stability (Crowther et al., 1998; Bertoncini et al., 2005). Furthermore, α-syn undergoes post-translational modifications of which one of the most common is phosphorylation of serine 129 (S129), which is found only in pathological α-syn deposits (Fujiwara et al., 2002; Doppler et al., 2014). S129 phosphorylation of recombinant α-syn has been reported to accelerate polymerization and promote aggregation (Fujiwara et al., 2002; Sano et al., 2018). It has also been reported that pathological changes are suppressed by inhibiting this phosphorylation in animal models. Thus, S129 phosphorylation is thought to have a significant effect on α-syn aggregation.

From another perspective, when nearly 40% of the volume is occupied by RNA and proteins, the intracellular environment becomes extremely crowded and water activity reduces (Gnutt and Ebbinghaus, 2016; Rivas and Minton, 2016). In this situation, the protein structure becomes compact, which promotes aggregation (Cheng et al., 2018). This condition has been reported to promote aggregation of disease-associated proteins that include α-syn, FUS, and PrP (Murray et al., 2017; de Oliveira et al., 2019; Ray et al., 2020; Tange et al., 2021). Disruption of the interaction between the NAC region and C-terminal domain by the environment, mutations, or post-translational modifications has been considered to promote α-syn aggregation.

**FIGURE 1:**
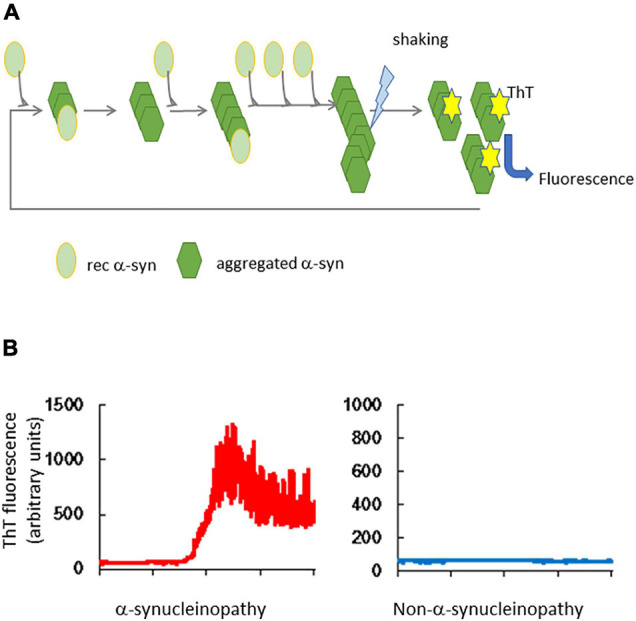
Real-time quaking-induced conversion of α-synuclein. **(A)** Outline of RT-QuIC. Tissues or body fluids of patients are added to a reaction buffer that contains recombinant α-synuclein (rec α-syn). Aggregated α-syn converts rec α-syn to the abnormal structure, which forms amyloid by repeated shaking and incubation. Thioflavin T (ThT) binds to the β-sheet structure of amyloid and its fluorescence is detected. **(B)** Results of α-syn RT-QuIC. The left panel shows the result of a brain homogenate from an α-synucleinopathy patient and the right panel shows the result of a non-α-synucleinopathy patient.

The QuIC assay is capable of detecting a very small amount of abnormal protein added as a seed to reaction buffer that contains a recombinant protein purified from *Escherichia coli* as the substrate; this is repeatedly and intermittently shaken and then left to stand. Thus, the products of the RT-QuIC reaction have a β-sheet-dominant structure from the α-helix-dominant structure, thereby promoting amyloid formation (Figure 1). Therefore, the structure of the substrates is induced to convert to the abnormal form by the QuIC reaction (Atarashi et al., 2008; Wilham et al., 2010). Thioflavin T (ThT), which is added to the reaction buffer, binds to amyloid and its fluorescence is measured at certain intervals to observe the amyloid formation reaction in real time (Atarashi et al., 2011).

The most commonly reported diagnostic method uses CSF of human prion patients (Atarashi et al., 2011; Sano et al., 2013; Foutz et al., 2017). Detection of abnormal aggregated α-syn in CSF of DLB patients by the RT-QuIC assay was first reported by Fairfoul et al. (2016); this was followed by multiple clinical studies. At present, diagnostic methods using skin (Orru et al., 2017) or OM (Orru et al., 2014; Bongianni et al., 2017), which includes CSF, have been reported (Satoh et al., 2017; Ascari et al., 2020; Candelise et al., 2020).

## Detection of Aggregated α-Synuclein in Cerebrospinal Fluid

Lumbar puncture is one of the most commonly performed procedures by neurologists. Therefore, RT-QuIC using spinal fluid has been widely studied as an effective method for early diagnosis of prion diseases (Atarashi et al., 2011; Sano et al., 2013; Foutz et al., 2017). CSF is also considered to be the most promising material for clinical diagnosis by α-syn RT-QuIC (Table 1).

**TABLE 1:** Sensitivity and specificity of α-syn RT-QuIC in CSF of patients.

Method	Substrate	α -Synucleinopathy			Number	Sensitivity (%)	Non-α -synucleinopathy			Number	Specificity (%)	References
1	WT	Total			32	93.8	Total			55	100	Fairfoul et al., 2016
				PD	20	95.0			AD	30	100	
				DLB	12	91.7			Healthy	20	100	
									CBD	3	100	
									PSP	2	100	
	WT	Neuropathologically verified cases										Bongianni et al., 2019
		Total (α-synucleinopathies)			28	92.9	Total (non-α-synucleinopathy)			49	95.9	
				DLB	7	100			sCJD	19	100	
				MSA-C	1	100			Other neurodegenerative diseases	11	90.9	
				LBD/AD	15	93.3						
				LBD/PART	2	100			Other neurological diseases	19	94.7	
				CJD/LBD	3	66.7						
		Clinical cases										
				Probable DLB	20	85.0			Probable AD	10	100	
				Possible DLB	6	0						
	WT	Total			85	75.3	Total			78	93.6	van Rumund et al., 2019
				PD	53	84.9			PSP	8	87.5	
				MSA	17	35.3			Tauopathy of uncertain origin	2	100	
				DLB	1	100			VaP	9	66.7	
				α-Synucleinopathy with vasculopathy	11	81.8			Other	7	100	
									Control	52	98.1	
				α-Synucleinopathy of uncertain origin	3	100						
	WT			PD	105	96.2			Healthy	79	82.3	Kang et al., 2019
	WT	Total of LRRK2 mutation			31	29.0						Garrido et al., 2019
				LRRK PD	15	40.0			Healthy control	10	20.0	
				LRRK NMC	16	18.8						
				IPD	10	90.0						
2	WT			PD	15	100			PSP	5	60.0	Manne et al., 2019
									No α-synucleinopathy	11	100	
2	WT	Total of definite cases			21	95.2	Total of definite cases			101	98.0	Rossi et al., 2020
				DLB	14	100			AD	17	94.1	
				Dementia with incidental LB	7	85.7			PSP	1	100	
									MSA	2	100	
									Syn-controls	81	98.8	
		Total of clinical cases			151	95.4	Total of clinical cases			166	94.0	
				DLB	34	97.1			AD	43	83.7	
				PD	71	94.4			Clinical controls	62	98.4	
									PSP/CBS	30	100	
									MSA	31	93.5	
2	K23Q			PD	12	91.7			AD	16	100	Groveman et al., 2018
				DLB	17	94.1			No α-synucleinopathy	15	100	
2	K23Q			PD	108	97.2			Healthy control	85	87.1	Orrù et al., 2020

*Method 1: temperature, 30°C; shaking speed, 200 rpm; shaking interval time, 1–14 min without SDS (except for Bongianni et al., 2019). Method 2: temperature, 42°C; shaking speed, 400 rpm; shaking interval time, 1–14 min; SDS in reaction buffer, 0.015%. PD, Parkinson’s disease; AD, Alzheimer’s disease; DLB, dementia with Lewy bodies; CBD, corticobasal degeneration; PSP, progressive supranuclear palsy; sCJD, sporadic Creutzfeldt–Jakob disease; MSA, multiple system atrophy; MSA-C, MSA with predominant cerebellar ataxia; LBD, Lewy body disease; PART, primary age-related tauopathy; VaP, vascular parkinsonism; LRRK2, leucine-rich kinase 2; NMC, non-manifesting carriers; IPD, idiopathic PD; CBS, corticobasal syndrome.*

In the first report of α-syn RT-QuIC, the authors investigated the amount of CSF used as the seed in pure DLB (*n* = 12) patients who were neuropathologically diagnosed in the OPTIMA cohort study compared with normal controls (Fairfoul et al., 2016). The sensitivity and specificity of pure DLB were 83 and 96%, respectively, when the volume of CSF was 5 μL. The sensitivity was increased up to 92% with 10 μL CSF and 98% with 15 μL, and the specificity of α-syn RT-QuIC was 100% when the seeds were both 10 and 15 μL CSF from patients clinically diagnosed with α-synucleinopathies. CSF obtained from patients with an unclear diagnosis of parkinsonism has been tested by α-syn RT-QuIC. The sensitivity and specificity were 75 and 94%, respectively (Table 1; van Rumund et al., 2019). This is the first report to examine a case in which no clinical diagnosis had been made. The lower sensitivity was thought to be because the test was performed in patients with an unclear diagnosis (only 50% of patients were diagnosed with α-synucleinopathy at 3 years after their lumber puncture). Then, a large cohort study of 439 clinically diagnosed and autopsied cases was performed. The sensitivity of 21 autopsied cases pathologically determined to be positive for LB-α-syn was 95.2% and the specificity in all 101 LB-α-syn-negative cases was 98% (Rossi et al., 2020). In the clinical cohort, the sensitivity was 95.4% and the specificity was 94% (Table 1).

Kang et al. (2019) compared the diagnostic accuracy of RT-QuIC (1 min shake at 200 rpm followed by 14 min rest) and PMCA in PD (1 min shake at 500 rpm followed by 29 min rest) patients from the BioFIND cohort and CSF from healthy controls. The sensitivity of RT-QuIC and PMCA in 105 PD patients was 96.2 and 95.2%, respectively, and the specificity of RT-QuIC and PMCA in the 79 healthy controls was 82.3 and 89.9%, respectively. The concordance rate between the two was as high as 92%, which indicated reliability and reproducibility of PD diagnosis.

Groveman et al. (2018) performed RT-QuIC at a higher temperature (30–42°C), higher shaking speed (200–400 rpm), and shorter incubation period (14→1 min) than the original assay and added 0.0015% SDS to the reaction buffer. They succeeded in shortening the lag phase from 50 to 20 h (Groveman et al., 2018). Furthermore, using a mutant α-syn (K23Q) as the substrate, they suppressed the slight increase in ThT fluorescence observed in healthy control CSF when WT α-syn was used as the substrate. The sensitivity of the assay using CSF of 12 PD and 17 DLB patients was 91.6 and 94.1%, respectively, and the specificity in 31 non-α-synucleinopathies, which included 16 AD cases, was 100%. They named this method rapid RT-QuIC (RT-QuICR) (Groveman et al., 2018). Additionally, they detected aggregated α-syn by RT-QuICR in the CSF of PD patients from the same BioFIND cohort as Kang et al. (2019) and compared the accuracy with the original RT-QuIC and PMCA. The specificity of RT-QuICR in 108 PD samples was 97.2%, and in 85 healthy controls, it was 87.1%, which was comparable with the other two assays (Orrù et al., 2020). The sensitivity and specificity of α-syn RT-QuIC using CSF for PD and DLB diagnoses have been sufficiently reliable in various studies (Table 1).

## Detection of Aggregated α-Synuclein in Familial Parkinson’s Disease Patients

Although all previous studies have focused on idiopathic α-synucleinopathy, a study on patients with the p.G2019S mutation in the leucine-rich kinase 2 (*LRRK2*) gene has also been reported. This mutation is the most frequently found in familial PD (Healy et al., 2008; Mancini et al., 2020). Garrido et al. (2019) tested patients with idiopathic PD (IPD), LRRK2-PD, or LRRK2 non-manifesting carriers (NMCs). IPD was positive in 9 of 10 patients (sensitivity 90%), whereas RT-QuIC positivity for LRRK2-PD was found in 6 of 15 patients (40%) and LRRK-NMC was found in only 3 of 16 patients (18.8%) (Table 1). Two of 10 healthy controls were positive (specificity 80%), and unlike IPD patients, LRRK2-PD patients had a lower rate of a positive reaction in the RT-QuIC assay, which the authors attributed to less insoluble synuclein and lower seed activity in the brain of LRRK2-PD patients compared with IPD patients. These data indicated that sensitivity for familial PD is lower than that for IPD. Further research is needed to increase the number of cases and to investigate other mutations.

## Immunostaining Analysis of α-Synuclein in Skin

Deposition of α-syn in dermal nerve fibers and decrease sof autonomic innervations of sweat glands, blood vessels, and erector pili muscles have been reported in PD patients. Wang et al. (2013) successfully detected native α-syn using an anti-α-syn antibody in dermal nerve fibers of PD patients and healthy controls. They also reported that the density of α-syn-positive nerve fibers in skin tissue was increased in PD patients compared with healthy subjects (Wang et al., 2013). Deposition of the disease-associated form, phosphorylated α-syn (p-α-syn), in dermal and epidermal fibers has also been reported. Gibbons et al. (2016) detected p-α-syn and quantified deposition of α-syn in the pilomotor or sudomotor of skin biopsies and found that α-syn deposition was higher in patients without autonomic symptoms than in healthy subjects (the sensitivity and specificity for PD compared with normal subjects was >90%). Other groups have reported that specific p-α-syn can be detected in skin biopsies by pathological findings (Ikemura et al., 2008; Donadio et al., 2016). The sensitivity and specificity of anti-phosphorylated synuclein antibodies have been analyzed by meta-analysis of 41 case–control studies that included 12 studies using skin tissues of patients (Tsukita et al., 2019). Three studies (Navarro-Otano et al., 2015; Donadio et al., 2016; Melli et al., 2018) (38 PD patients and 42 controls) using anti-α-syn antibodies showed 76% sensitivity and 60% specificity. In nine studies using anti-p-α-syn antibodies (170 PD patients and 214 controls) (Donadio et al., 2014, 2016, 2018b; Doppler et al., 2014, 2015, 2017; Navarro-Otano et al., 2015; Zange et al., 2015; Melli et al., 2018), the sensitivity was 76% and the specificity was 100% in a pathological approach (Table 2). Thus, the presence of α-syn in the skin tissue of PD patients has been widely demonstrated. The distribution of p-α-syn has been analyzed and compared between clinical variants of α-synucleinopathy. Donadio et al. (2016, 2018b) compared positive rates of p-α-syn in cervical, thigh, and leg (malleolus) regions. In PD and DLB patients, all cervical regions were positive, but the positivity rate was decreased in the order of the thigh and leg. Conversely, in MSA patients, the leg had the highest positivity rate at about 70% (40% in PD) and the positivity rate was decreased in the order of thigh and cervical regions, which was the opposite trend to PD and DLB. Patients with pure autonomic failure (PAF), which is a prodromal stage of synucleinopathies, had 100% positivity in all three sites. In a comparison of skin tissues from PD and DLB patients without OH, the most sensitive site was the central region (especially the posterior cervical region). However, in cases with OH, p-α-syn is widely distributed, and in the early stage of the disease, p-α-syn is detected first in the feet (Donadio et al., 2014, 2018a; Doppler et al., 2015; Niemann et al., 2021). These reports suggest that the site of the skin biopsy and the symptoms of patients have a significant effect on differential diagnosis of synucleinopathies.

**TABLE 2:** Sensitivity and specificity of the detection of abnormal α-synuclein aggregation in skin tissues.

Method	Seed/antibody	Sample	α -Synucleinopathy	Number	Sensitivity (%)	Non-α -synucleinopathy	Number	Specificity (%)		References
										
RT-QuIC	Homogenate	Autopsy	PD	18	100	Healthy control	25	96.0		Manne et al., 2020
	FFPE		PD	28	92.9	Healthy control	12	83.3		
	Emulgion (abdominal)	Autopsy	Total (α-synucleinopathies)	57	93.0	Total (non-α-synucleinopathy)	73	91.8		Wang et al., 2021
			PD	47	93.6	Non-neurodegenerative control	43	97.7		
			LBD	7	100	AD	17	70.6		
			MSA	3	66.7	CBD	5	100		
						PSP	8	100		
	Homogenate (posterior cervical)	Biopsy	PD	20	95.0	Non-PD	21	100		
IF	Anti-n-α-syn antibody	Biopsy	PD	38	76.3	Healthy control	42	59.5	Pooled data of three studies	Tsukita et al., 2019
	Anti-p-α-syn antibody	Biopsy	PD	170	75.9	Healthy control	214	100	Pooled data of nine studies	

*Anti-n-α-syn antibody, anti-normal α-syn antibody; Anti-p-α-syn antibody, anti-phosphorylated α-syn antibody; FFPE, formalin fixed paraffin embedded.*

## Detection of Aggregated α-Synuclein in the Skin by Real-Time Quaking-Induced Conversion

Manne et al. (2020) attempted to detect aggregated α-syn by RT-QuIC in frozen tissue and in formalin paraffin-embedded (FFPE) sections of occipital skin tissue from PD patients diagnosed by autopsy (Table 2). Frozen tissues from 24 of 25 PD patients and 1 of 25 healthy controls were positive in the RT-QuIC assay (sensitivity: 96%; specificity: 96%). In FFPE sections, 9 of 12 PD patients and 2 of 12 healthy controls were positive (sensitivity: 75%; specificity: 83%). The reasons for the low sensitivity and specificity in FFPE sections are thought to be that the amount of tissue used for the test was limited and the seeding activity was suppressed by formalin fixation.

Wang et al. (2021) analyzed abdominal skin of 130 cadavers with PD, DLB, MSA, AD, PSP, or CBD by the α-syn RT-QuIC assay. They detected aggregated α-syn in 44 of 47 PD samples, and all 7 DLB samples and 2 of 3 MSA samples were positive (sensitivities of 94, 100, and 67%, respectively; Table 2; Wang et al., 2021). The combined sensitivity of the three α-synucleinopathies was 93%. The specificity for NNC, PSP, CBD, and AD combined was 93% and that for NNC alone was 98%. The authors also used PMCA (substrate was mouse brain homogenate and induced conversion by sonication) (Nicot et al., 2019) to detect aggregated α-syn in the same samples. The sensitivity and specificity to distinguish α-synucleinopathies from non-α-synucleinopathies were 82 and 96%, respectively. The concordance rate for PD was 78.6%, which was slightly higher than that using RT-QuIC, but there was no significant difference in the McNemar test. The authors noted that the accuracy of both detection methods was about the same. Additionally, the authors compared the sensitivity and specificity of these two methods for detection of aggregated α-syn in the skin of living patients. The sensitivity and specificity of RT-QuIC were 95 and 100%, respectively, whereas those of PMCA were 80 and 90%, respectively, which is consistent with reports that the positive rate of aggregated α-syn in the RT-QuIC assay is higher in skin samples near the center of the body than in other skin samples.

The sensitivity and specificity of α-syn RT-QuIC using autopsy and biopsy samples are comparable with those using CSF, but the control group in the study using biopsy samples comprised healthy controls. Because all seven DLB autopsy cases were positive, it is possible that biopsies can also be used.

Wang et al. (2021) also performed RT-QuIC using leg lesions from skin biopsies of PD patients. In contrast to the cervical region, which showed an increase in ThT fluorescence within 30 h, the leg skin tissue showed a weaker aggregation response and required more than 50 h to show a positive signal. This is the same result as detection of p-α-syn by immunostaining. Thus, the skin collection site greatly affects the accuracy of diagnosis (Tsukita et al., 2019). These results led to the conclusion that examination of skin samples from appropriate sites may be helpful to differentiate diseases.

One study compared the accuracy of skin immunofluorescence (IF), skin RT-QuIC, and CSF RT-QuIC in diagnosing α-synucleinopathies (Donadio et al., 2021). The sensitivity and specificity of skin IF were 90 and 100%, those of skin RT-QuIC were 86 and 80%, and those of CSF RT-QuIC were 78 and 100%, respectively. These results suggest that skin IF is the most reliable. However, the sensitivity of skin IF in nine reports of skin IF analyzed by Tsukita et al. (2019) was low (76%; Table 2). Moreover, the total sensitivity of CSF RT-QuIC for both PD and DLB as shown in Table 1 was 94% and the specificity for AD and healthy controls was 93%. Therefore, the sensitivity of CSF RT-QuIC appears to be higher than that of skin IF. Currently, it is unclear which method is better, but it is important to at least combine these methods to take advantage of their characteristics.

### Detection of Aggregated α-Synuclein in Cerebrospinal Fluid of Multiple System Atrophy Patients

Detection of aggregated α-syn in the CSF of MSA patients was conducted in two studies. The sensitivities were 6.5% (Rossi et al., 2020) and 35% (van Rumund et al., 2019), which are much lower than those for AD and DLB. Structural differences in the fibrils of aggregated α-syn between MSA and PD have been suggested as the reason for the low sensitivity (Bousset et al., 2013; Peelaerts et al., 2015; Shahnawaz et al., 2020), which is similar to the differences in the biochemical properties of prion strains (Sano et al., 2015; Morales, 2017).

De Luca et al. (2019) attempted to detect aggregated α-syn by RT-QuIC using OM of MSA patients. RT-QuIC using OM samples is also a successful method for prion detection (Bongianni et al., 2017). OM samples were collected from clinically diagnosed PD and MSA patients and RT-QuIC was performed. Ten of 18 (56%) PD patients were positive, whereas 9 of 11 (82%) MSA patients were positive. Additionally, 1 of 6 and 2 of 12 patients were positive for CBD and PSP, respectively. RT-QuIC using OM samples has the potential to be a diagnostic tool for MSA, although large-scale studies are needed. The α-syn-PMCA (1 min shake at 500 rpm followed by 29 min rest) assay discriminates between CSF samples from PD and MSA patients with an overall sensitivity of 95.4%. They used a combination of biochemical, biophysical, and biological methods to analyze the product of α-syn-PMCA and found that the characteristics of the α-syn aggregates in CSF can be used to readily distinguish between PD and MSA. They also found that the properties of aggregates amplified from CSF were similar to those from the brain. Furthermore, there are structural differences between α-syn aggregates derived from patients with PD or MSA in Cryo-ET (Shahnawaz et al., 2020).

## Detection of Aggregated α-Synuclein in Patient Samples at the Prodromal Stage of α-Synucleinopathies

Idiopathic/isolated rapid eye movement (REM) sleep behavior disorder (iRBD) is a disorder characterized by behavioral abnormalities during REM sleep (Sateia, 2014; St Louis et al., 2017). PAF is a sporadic, slow progressive disorder that develops in adulthood and is clinically characterized by OH with a tendency to faint. The clinical features are OH with a tendency for syncope (No authors listed, 1996; Coon et al., 2019). These disorders are strong early signs of α-synucleinopathies.

α-Synuclein aggregates have been detected in CSF from 18 iRBD and 28 PAF patients with sensitivities of 100 and 92.9%, respectively (Table 3; Rossi et al., 2020), whereas all 3 RBD patients in the first report of α-syn RT-QuIC were negative (Fairfoul et al., 2016). A large cohort study of iRBD patients has also been reported. α-Syn RT-QuIC was performed using the CSF of 52 iRBD patients and 40 healthy controls to investigate whether aggregated α-syn can be a biomarker for prodromal α-synucleinopathy in a long-term follow-up after spinal fluid collection (Table 3; Iranzo et al., 2021). Forty-seven patients (90%) in the iRBD group and four patients (10%) in the healthy control group were positive. Thirty-two patients developed PD or DLB after a mean of 3.4 years, and among them, 31 patients (97%) were positive in RT-QuIC (Table 3).

**TABLE 3:** Sensitivity and specificity of RT-QuIC in patients at the prodromal stage of α-synucleinopathy.

Substrate	α -Synucleinopathy	Number	Sensitivity (%)	Non-α -synucleinopathy	Number	Specificity (%)	References
CSF	Risk for PD (RBD)	3	0	See Table 1			Fairfoul et al., 2016
	iRBD	18	100	See Table 1			Rossi et al., 2020
	PAF	28	92.9				
	Total iRBD	52	90.4	Total non-iRBD	51	92.2	Iranzo et al., 2021
	Disease free	20	80.0	Healthy control	40	90.0	
	Converted to PD	16	93.8	Autosomal dominant AD	5	100	
	Converted to DLB	16	100	Narcolepsy type 1	6	100	
OM	iRBD	63	44.4	Healthy control	10	89.8	Stefani et al., 2021
	PD	41	46.3				

*iRBD, idiopathic/isolated rapid eye movement (REM) sleep behavior disorder; PAF, pure autonomic failure.*

Detection in OM samples has also been reported. Twenty-eight of 63 (44.4%) iRBD patients and 19 of 41 (46.3%) PD patients were positive (Stefani et al., 2021). The sensitivity in PD patients was similar to that reported by De Luca et al. (2019; Table 3). Although the sensitivity was not high, this result suggests that aggregated α-syn is already present in OM at the iRBD stage. RT-QuIC using skin has not yet been reported, whereas p-α-syn has been detected by IF in 23 of 28 iRBD patients without PD or DLB (Al-Qassabi et al., 2020). Therefore, RT-QuIC using skin tissues of iRBD patients may also be possible.

It has been reported that >90% of patients develop α-synucleinopathy within 14 years of diagnosis of iRBD (Iranzo et al., 2013, 2014) and 24–34% of PAF patients develop α-synucleinopathies (Mabuchi et al., 2005; Kaufmann et al., 2017; Singer et al., 2017; Coon et al., 2020). It has been suggested that patients who are positive in RT-QuIC at the time of iRBD diagnosis have a high probability of developing α-synucleinopathy. Therefore, the establishment of RT-QuIC for patients with iRBD and PAF may enable prediction of the onset of α-synucleinopathies and therapeutic intervention before the onset.

### Differentiation of Patients With Rapidly Progressive Dementia

Sano et al. (2018) performed α-syn RT-QuIC on autopsied brain samples and found that all seven DLB cases were positive, whereas all CJD and AD cases were negative. RT-QuIC has also been performed using CSF from 77 autopsied cases of suspected CJD, which showed positive results in all cases (100% sensitivity) and 18 of 20 other neurodegenerative diseases with α-synucleinopathy co-pathology were positive (90% sensitivity) (Bongianni et al., 2019). The combined sensitivity for all α-synucleinopathies was 92.9%. Additionally, only 2 of 49 neuropathological diagnoses of non-α-synucleinopathies, which included 19 sporadic CJD, were positive, with a specificity of 95.9% (Table 1). These reports indicate that α-syn RT-QuIC can be used to accurately differentiate DLB from CJD-suspected cases (Rossi et al., 2020), but the number in this study and the standardization of α-syn RT-QuIC were insufficient. We assumed that α-syn RT-QuIC was not capable of differentiating DLB from CJD in the last few years, but α-syn RT-QuIC, prion-QuIC, and tau-QuIC assays can differentiate DLB from other rapidly progressive dementias (Sano et al., 2018).

## Discussion

### False Positive Is False?

In many clinicopathological studies conducted to date, healthy controls, and AD, PSP, and CBD patients were included as negative control groups, which reported some “false positive” results. Cross-seeding of pathological proteins, such as tau and amyloid β, has been reported *in vitro* and in animal models, which acted as the seed for α-syn (Clinton et al., 2010; Candelise et al., 2019). To resolve this problem, it is necessary to find a substrate or seed that does not cause cross-seeding. Mixed pathologies can be caused by cross-seeding. In a clinical study, it was reported that 88 of 147 patients diagnosed with AD were positive for α-syn (Ronald, 2000). Fairfoul et al. (2016) reported a 65% positive rate in patients with mixed AD and LBD, and 15% in patients with AD and LB. If such cases are clinically diagnosed as AD, they may be the cause of false positives.

## The Limit of the α-Synuclein Real-Time Quaking-Induced Conversion Assay

Many researchers, ourselves included, are developing methods for the α-syn RT-QuIC assay. Evidence is also being established to show that the α-syn RT-QuIC method is particularly useful to diagnose DLB and MSA-P in α-synucleinopathies. However, there are currently no biomarkers that reflect the clinical time course during clinical care. In particular, the development of synuclein PET is progressing, but there is still no ideal probe that can be advanced to clinical trials. Even if the development of α-syn RT-QuIC progresses, α-syn RT-QuIC has difficulty in reflecting chronological biomarkers. α-Syn RT-QuIC is useful for diagnosis, but α-syn RT-QuIC does have a biomarker that reflects chronological biomarkers. We believe that this is a limit of the α-syn RT-QuIC assay.

## Conclusion

Parkinson’s disease and DLB can be diagnosed with high accuracy by α-syn RT-QuIC, but the sensitivity and specificity of α-syn RT-QuIC may be inferior to that of prion RT-QuIC. This may be related to LBs not having a high disease specificity compared with abnormal PrP deposition in human prion diseases. In some studies on α-syn RT-QuIC, patients who were diagnosed by clinical symptoms were tested. The accuracy of the clinical diagnosis of PD has been reported to be about 80% (Adler et al., 2014; Marsili et al., 2018). Studies on RT-QuIC with follow-up and autopsy are indispensable to improve accuracy, although they are difficult because the disease course of α-synucleinopathies is longer than that of CJD.

To establish diagnosis by RT-QuIC, it is desirable to develop a method that does not cause cross-seeding, a detection method employing body fluids that can be collected easier (e.g., tears, sweat, and saliva), and a method to differentiate the type of α-synucleinopathy.

## Author Contributions

KS supervised the study and wrote the manuscript. NN and TN collected information, wrote the manuscript, and participated in discussions. All authors contributed to the article and approved the submitted version.

## Conflict of Interest

The authors declare that the research was conducted in the absence of any commercial or financial relationships that could be construed as a potential conflict of interest.

## Publisher’s Note

All claims expressed in this article are solely those of the authors and do not necessarily represent those of their affiliated organizations, or those of the publisher, the editors and the reviewers. Any product that may be evaluated in this article, or claim that may be made by its manufacturer, is not guaranteed or endorsed by the publisher.
